# Cardiovascular Safety During and After Use of Phentermine and Topiramate

**DOI:** 10.1210/jc.2018-01010

**Published:** 2018-09-21

**Authors:** Mary E Ritchey, Abenah Harding, Shannon Hunter, Craig Peterson, Philip T Sager, Peter R Kowey, Lan Nguyen, Steven Thomas, Miguel Cainzos-Achirica, Kenneth J Rothman, Elizabeth B Andrews, Mary S Anthony

**Affiliations:** 1 RTI Health Solutions, Research Triangle Park, North Carolina; 2 VIVUS, Inc., Campbell, California; 3 Stanford University School of Medicine, Stanford, California; 4 Sidney Kimmel Medical College, Thomas Jefferson University, Philadelphia, Pennsylvania; 5 RTI Health Solutions, Barcelona, Spain; 6 RTI Health Solutions, Waltham, Massachusetts

## Abstract

**Context:**

Increases in heart rate were seen during the clinical program for fixed-dose combination phentermine (PHEN) and topiramate (TPM), an oral medication indicated for weight management; however, the effect on cardiovascular (CV) outcomes is uncertain.

**Objective:**

The aim of the present study was to determine the extent to which the rates of major adverse CV events (MACE) in patients using PHEN and TPM (including fixed dose) differed from the MACE rates during unexposed periods.

**Design:**

Retrospective cohort study.

**Setting:**

MarketScan, US insurance billing data.

**Patients or Other Participants:**

Patients aged >18 years with ≥6 months of continuous enrollment in the database before taking PHEN and/or TPM or after stopping these medications.

**Interventions:**

PHEN and TPM, taken separately and together (including fixed dose).

**Main Outcome Measures:**

MACE, a composite of hospitalization for acute myocardial infarction and stroke and in-hospital CV death.

**Results:**

Because the outcomes are rare and the duration of medication use was brief, few events occurred. The MACE rates among current users of PHEN/TPM, fixed-dose PHEN/TPM, and PHEN were lower than those among unexposed former users. In contrast, the rate of MACE among current users of TPM was greater than among unexposed former users [incidence rate ratio: PHEN/TPM, 0.57; 95% CI, 0.19 to 1.78; fixed-PHEN/TPM, 0.24; 95% CI, 0.03 to 1.70; PHEN, 0.56; 95% CI, 0.34 to 0.91; TPM, 1.58; 95% CI, 1.33 to 1.87).

**Conclusions:**

Overall, the data indicated no increased risk of MACE for current PHEN/TPM users; however, the 95% CIs for the PHEN/TPM groups were broad, indicating that the data were compatible with a wide range of possible values.

## Teaser

In the present retrospective cohort safety study, the current users of combination phentermine and topiramate did not have an increased incidence rate of MACEs compared with former users.

Nearly 40% of adults in the United States are obese. The prevalence is even greater among older adults and has been increasing during the past 15 years (1). Obese individuals have greater mortality rates than the general population and an increased risk of overall, cardiovascular (CV)-related, and diabetes-related mortality (2). In recent years, several medications have received US Food and Drug Administration (FDA) approval as adjuncts to diet and lifestyle modifications for the management of obesity. One such medication is a fixed-dose combination of phentermine and extended-release topiramate (Qsymia^®^, Vivus, Inc.). Phentermine (PHEN) is a stimulant and is indicated for short-term use in weight management. It acts as an appetite suppressant via the central nervous system. Topiramate (TPM) is an anticonvulsant indicated for use in the treatment of migraine and epilepsy. One of the known effects of TPM is a decrease in appetite. Consequently, it is sometimes prescribed off-label for weight loss (3). Typical dosing of PHEN is 15 to 37.5 mg/d. The typical dosage of TPM is 100 mg/d for migraine or 50 to 400 mg/d for epilepsy. The approved fixed-dose combination (fixed-PHEN/TPM) contains PHEN doses from 3.75 to 15 mg and TPM doses from 23 to 92 mg for daily administration.

The results from two randomized clinical trials and one 2-year extension study showed a slight increase in the average heart rate for those taking fixed-PHEN/TPM (4–6). For those taking high-dose fixed-PHEN/TPM (PHEN 15 mg/TPM 92 mg), average heart rate increased from baseline by 1.2 to 1.7 beats per minute. In these same trials, several traditional CV risk factors were improved with fixed-PHEN/TPM treatment, specifically decreased body weight and body mass index, lower systolic and diastolic blood pressure, lower low-density lipoprotein cholesterol and plasma triglyceride concentrations, and lower fasting blood glucose levels.

These studies provided limited information on the CV safety of fixed-PHEN/TPM and its component medications as they are currently used within clinical practice. Therefore, a randomized, prospective postmarketing outcome study of major adverse CV events (MACE) was requested by a regulatory agency. However, usage of fixed-PHEN/TPM is low, and performance of a randomized study of medications in a postmarket setting is difficult, especially for CV event outcomes. With low drug uptake and rare outcomes, a retrospective observational database study is an efficient method to generate information on the safety of fixed-PHEN/TPM in usual clinical practice in a much shorter time than would be possible with a prospective study.

**Figure 1.**
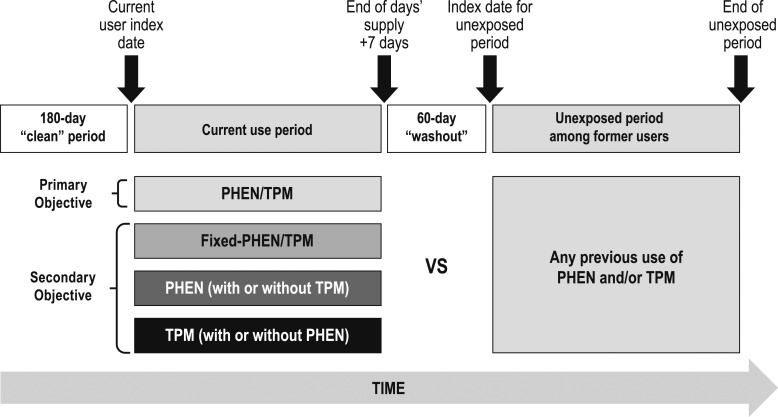
Schematic of cohorts with comparisons indicated.

The aim of the present study was to evaluate the risk of MACE during current use periods of PHEN, TPM, PHEN/TPM (including the two drugs separately and in a fixed-dose combination), and fixed-PHEN/TPM (only the fixed-dose combination) vs unexposed periods among former users of PHEN, TPM, or both PHEN and TPM (Fig. 1).

## Subjects and Methods

### Study design and population

The present retrospective cohort study was conducted in the Truven Health MarketScan Databases (Commercial and Medicare Supplemental administrative claims). MarketScan was chosen as the data source because it had the largest number of fixed-PHEN/TPM users among the databases evaluated during an earlier feasibility assessment. In addition, it has a suitable breadth of information available about patients and the ability to capture the medication exposures and CV outcomes reliably. A prospective study with the number of patients available in the present database would not be feasible because of the large study size and long duration required for CV outcome studies.

**Figure 2.**
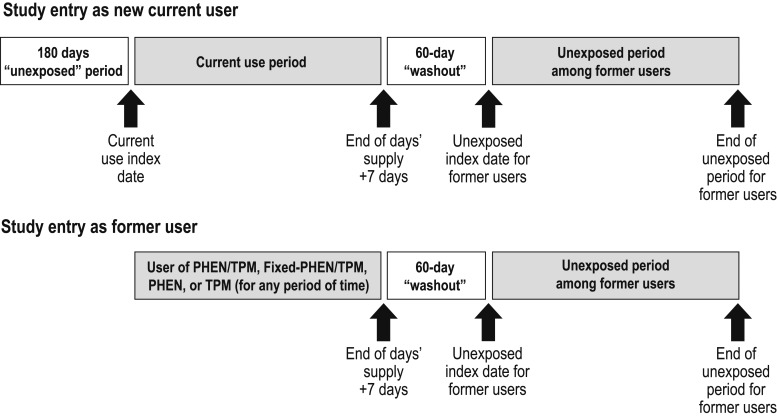
Schematic of risk periods.

The data span the period beginning 1 July 2012, the month Qsymia was approved by the US FDA, through 30 September 2015. Patients were eligible for study entry if they were aged ≥18 years and had been enrolled in MarketScan for ≥6 months and met the criteria to be included as a current or former user of PHEN, TPM, PHEN/TPM, and/or fixed-PHEN/TPM (Fig. 2). Depending on their medication use, patients could simultaneously contribute time to more than one of these variously defined current-use medication cohorts. For example, patients prescribed fixed-PHEN/TPM contributed to the current-use fixed-PHEN/TPM, PHEN, TPM, and PHEN/TPM medication cohorts simultaneously.

We used the periods corresponding to current use of medications as the exposed time at risk. We compared the rates during those periods with the rates among the unexposed periods among former users of the study medications. Because these cohorts are dynamic, with patients moving in and out of them, a given patient could contribute to both current use of medication and, when no longer taking the medication, to the unexposed time at risk. However, not all patients contributed to both current-use and unexposed periods. The index date for current-use periods was the date of the first prescription dispensed after a ≥180-day period free of exposure (for the initial entry into the cohort) or after a gap of >60 days (for subsequent use). The index date for unexposed periods was the first day on which eligibility criteria were met and >60 continuous days without exposure to any of the study medications. Patients could contribute time to multiple current-use periods and unexposed periods if they had started, stopped, or switched study medications. Patients were excluded if they had undergone a surgical procedure for weight loss or dispensing of fenfluramine or dexfenfluramine before their first index date. In addition, because TPM is indicated for seizures and epilepsy, we excluded patients who had been dispensed TPM without PHEN if they had a diagnosis for seizures or epilepsy within 30 days before the initial TPM prescription, or if they had been prescribed daily doses of >100 mg of TPM (doses associated with epilepsy).

The decision to compare outcomes during periods of current exposure with those during unexposed periods among former users of these same medications rather than with nonusers was determined from two major considerations. First, because the underlying condition of obesity or overweight status is often not captured with the diagnosis codes in claims data, it is difficult to match a nonuser cohort to current users of these drugs by obesity status. Using nonexposed periods in former users as a comparison achieves partial balance for obesity status. Second, because the signal of potential concern with Qsymia is increased heart rate—an effect that does not persist after the drug is withdrawn—the potential for carryover effects beyond current use was considered remote.

### Key variables

The current-use periods of each medication began on the index date of the dispensing of that medication and continued until 7 days after the end of the last days’ supply of the last dispensing (*i.e.,* 37 days after the last prescription fill). Unexposed periods began 60 days after the end of the days’ supply of the last medication dispensing and continued until a new study medication was dispensed or the end of patient follow-up.

Outcomes were defined by the hospital admission and principal diagnosis codes using *International Classification of Diseases, Ninth Revision, Clinical Modification* (ICD-9-CM). Acute myocardial infarction (AMI) was defined using ICD-9-CM codes 410.x0 and 410.x1, and stroke was defined using ICD-9-CM codes 430, 431, 433.x1, 434 (excluding 434.x0), and 436 (7). These codes have been validated in claims databases with positive predictive values ranging from 76% to 94% (7, 8). In-hospital CV-related death was identified using the discharge status “died” and either a principal discharge diagnosis of AMI, stroke, heart failure, coronary heart disease, or cerebrovascular disease or a procedure code during the hospitalization indicating CV revascularization. The composite endpoint of hospitalization for AMI or stroke and in-hospital CV-related death was MACE. Out-of-hospital death was not available from the data source and was thus not included in the present study.

Covariates were assessed across all available look-back time, which was ≥180 days before each index date (9). Covariates included age at index date, sex, and hospitalization for CV disease, length of hospitalization, duration of look-back time, comorbidities defined by diagnoses, and history of medication use. Comorbidity diagnoses were assessed via ICD-9-CM codes and included obesity, previous AMI, previous stroke, transient ischemic attack, hypertension, heart failure, unstable angina, peripheral vascular disease, coronary heart disease, cerebrovascular disease, hyperlipidemia, prediabetes, diabetes mellitus, chronic kidney disease, migraine, and sleep apnea. Medication history was defined using National Drug Codes and the following medications were included as covariates: antihypertensive agents, lipid-modifying agents, anticoagulants, other CV drugs (*e.g.,* vasodilating agents), insulin, other antidiabetic drugs, antiobesity drugs other than the ones included in the study, epilepsy drugs (other than TPM), migraine drugs (other than TPM), and/or prescription aspirin. In addition, the calendar year and month of the index date were collected.

### Statistical analysis

Descriptive statistics for demographic variables and relevant covariates were obtained for each current-use medication cohort (PHEN/TPM, fixed-PHEN/TPM, PHEN, and TPM) and for the unexposed former-user cohort.

The crude incidence rates, crude and adjusted incidence rate ratios (IRRs), and crude and adjusted incidence rate differences (IRDs) for each study outcome were calculated separately for current-use periods of each medication and for the unexposed periods.

Propensity score methods were used to control for confounding. Three separate propensity score models were developed for comparison of current use of each of the four medication/combinations [PHEN/TPM (including fixed-PHEN/TPM), PHEN, and TPM] vs unexposed former users. Propensity score models were created by assessing the effects of each potential covariate on the composite MACE outcome. The balance of covariates between current users and the unexposed was assessed using standardized differences. Subjects with extreme propensity scores <2.5th or >97.5th percentile were trimmed before stratification into deciles based on the distribution of current users. Stratum-specific IRRs and IRDs were calculated and summary IRR and IRD were calculated using the Mantel-Haenszel approach outlined in Rothman *et al.* (10).

We conducted sensitivity and bias analyses to determine whether the choices made for variable definitions and the comparator group were affecting the results. Sensitivity analyses were conducted for cohorts in which ≥10 MACE outcomes had occurred during the current-use periods. Analyses included assessing the effect of potential unmeasured confounders (*e.g.,* smoking) via proxy variables (*e.g.,* diagnosis of chronic obstructive pulmonary disease), assessing an alternative outcome of MACE that includes hospitalization for heart failure, limiting the length of the current-use periods and unexposed periods to a maximum of 6 months, requiring 180 days between prescriptions to initiate a subsequent current-use period, assuming that only a proportion of CV-related deaths (*e.g.,* 30%) was captured during hospitalizations (in contrast to deaths occurring outside the hospital) for current user cohorts. In addition, current users vs the unexposed were assessed in mutually exclusive medication cohorts (*e.g.,* comparing current-use periods of PHEN/TPM to unexposed periods among former users of PHEN/TPM).

## Results

### Patient demographics

**Figure 3.**
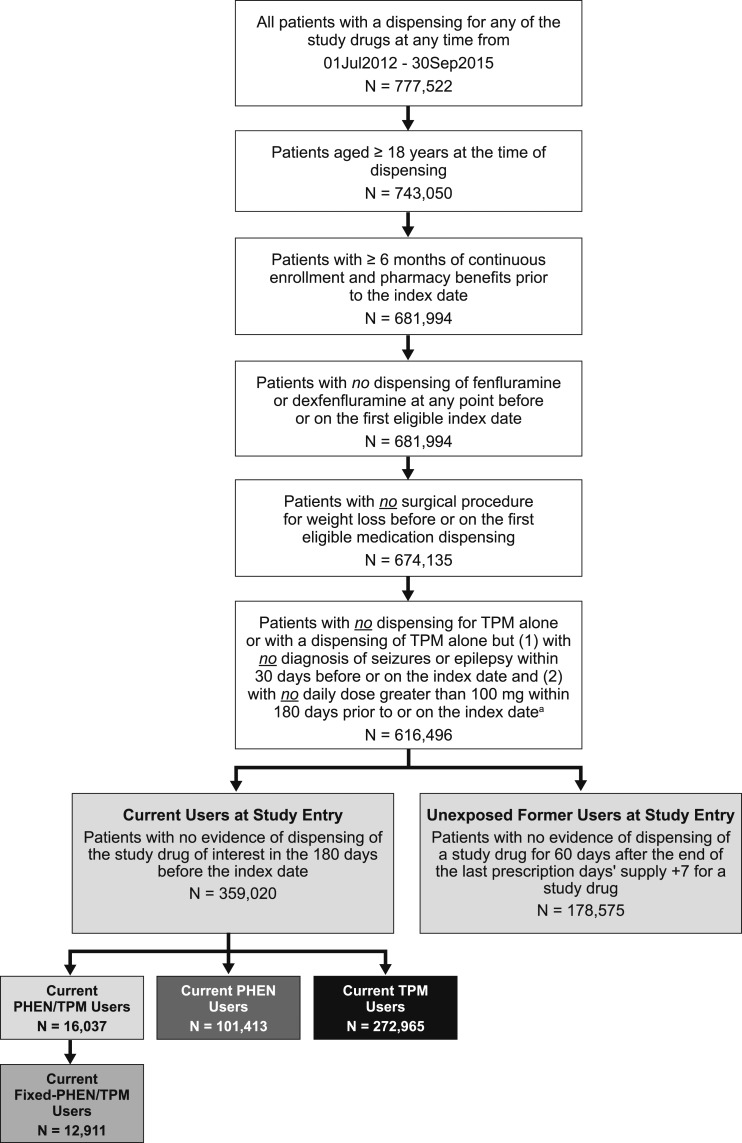
Flow diagram of selection into study cohorts.

Patients included and excluded from the present study are shown in Fig. 3. The characteristics for each patient at the first entry into each current-use medication cohort and into the unexposed cohort are listed in Table 1. More than 500,000 patients were included in the present study; 14,586 contributed time at risk to the fixed-PHEN/TPM cohort and an additional 4598 to the cohort of PHEN/TPM as individual medications. A single patient could be included in multiple cohorts, corresponding to multiple columns in Table 1. On average, patients contributed 1.6 current-use periods within a single medication cohort and 2.6 unexposed periods. There were 16,365 current-use periods, averaging 1.9 months among patients taking fixed-PHEN/TPM; 21,405 current-use periods, averaging 1.9 months among patients taking PHEN/TPM; 165,737 current-use periods, averaging 1.7 months for PHEN; 373,753 current-use periods, averaging 2.1 months for TPM; and 472,630 unexposed periods, averaging 7.9 months.

**Table 1.** Baseline Patient Demographics, Medical Comorbidities, and Medications

Characteristic	Current-Use Periods				Unexposed (n = 386,136)
	PHEN/TPM (n = 19,184)	Fixed-PHEN/TPM (n = 14,586)	PHEN (n = 124,334)	TPM (n = 316,388)	
Age, y	46.5 ± 10.94	47.3 ± 10.81	43.8 ± 11.22	43.2 ± 13.32	43.7 ± 13.00
Age categories, n (%)					
18–37 y	4161 (21.7)	2868 (19.7)	37,691 (30.3)	109,988 (34.8)	127,586 (33.0)
38–49 y	7093 (37.0)	5270 (36.1)	46,482 (37.4)	102,348 (32.3)	127,824 (33.1)
≥50 y	7930 (41.3)	6448 (44.2)	40,161 (32.3)	104,052 (32.9)	130,726 (33.9)
Sex, n (%)					
Male	3747 (19.5)	2964 (20.3)	21,358 (17.2)	55,765 (17.6)	66,541 (17.2)
Female	15,437 (80.5)	11,622 (79.7)	102,976 (82.8)	260,623 (82.4)	319,595 (82.8)
Medical history and comorbid conditions, n (%)					
Obesity	10,066 (52.5)	8147 (55.9)	45,339 (36.5)	72,451 (22.9)	104,657 (27.1)
AMI	54 (0.3)	45 (0.3)	267 (0.2)	1831 (0.6)	1983 (0.5)
Stroke	141 (0.7)	111 (0.8)	688 (0.6)	8595 (2.7)	9378 (2.4)
Transient ischemic attack	150 (0.8)	116 (0.8)	745 (0.6)	7575 (2.4)	8278 (2.1)
Hypertension	8529 (44.5)	6832 (46.8)	41,659 (33.5)	107,386 (33.9)	130,473 (33.8)
Heart failure	213 (1.1)	176 (1.2)	950 (0.8)	5626 (1.8)	6309 (1.6)
Unstable angina	152 (0.8)	124 (0.9)	663 (0.5)	3473 (1.1)	3803 (1.0)
Peripheral vascular disease	551 (2.9)	456 (3.1)	2037 (1.6)	8528 (2.7)	9940 (2.6)
Coronary heart disease	997 (5.2)	839 (5.8)	4170 (3.4)	18,672 (5.9)	21,440 (5.6)
Cerebrovascular disease	733 (3.8)	583 (4.0)	3201 (2.6)	24,681 (7.8)	28,070 (7.3)
Hyperlipidemia	8927 (46.5)	7197 (49.3)	43,124 (34.7)	107,087 (33.8)	132,853 (34.4)
Prediabetes	1703 (8.9)	1430 (9.8)	6936 (5.6)	17,031 (5.4)	20,439 (5.3)
Diabetes	3974 (20.7)	3315 (22.7)	15,741 (12.7)	39,957 (12.6)	48,405 (12.5)
Chronic kidney disease	340 (1.8)	284 (1.9)	1299 (1.0)	6167 (1.9)	7890 (2.0)
Sleep apnea	3205 (16.7)	2652 (18.2)	12,733 (10.2)	36,389 (11.5)	44,237 (11.5)
Migraine	1914 (10.0)	1261 (8.6)	11,165 (9.0)	124,960 (39.5)	159,307 (41.3)
Epilepsy	99 (0.5)	71 (0.5)	636 (0.5)	6035 (1.9)	14,704 (3.8)
Medication history, n (%)					
Antihypertensive agents	9283 (48.4)	7340 (50.3)	46,755 (37.6)	133,538 (42.2)	163,447 (42.3)
Lipid-modifying agents	5499 (28.7)	4516 (31.0)	24,185 (19.5)	67,373 (21.3)	82,201 (21.3)
Anticoagulant agents	808 (4.2)	681 (4.7)	3528 (2.8)	15,433 (4.9)	17,996 (4.7)
Other CV system drugs	2 (0.0)	2 (0.0)	7 (0.0)	62 (0.0)	68 (0.0)
Insulin	945 (4.9)	833 (5.7)	2968 (2.4)	8712 (2.8)	9835 (2.5)
Other antidiabetic drugs	4319 (22.5)	3541 (24.3)	16,883 (13.6)	36,417 (11.5)	45,103 (11.7)
Other antiobesity drugs	2851 (14.9)	2293 (15.7)	13,989 (11.3)	40,640 (12.8)	39,867 (10.3)
Aspirin	79 (0.4)	68 (0.5)	315 (0.3)	1210 (0.4)	1465 (0.4)
Migraine drugs	1645 (8.6)	1185 (8.1)	9233 (7.4)	93,252 (29.5)	116,409 (30.1)
Epilepsy drugs	2663 (13.9)	2032 (13.9)	14,917 (12.0)	84,545 (26.7)	100,743 (26.1)
TPM	2010 (10.5)	672 (4.6)	7789 (6.3)	56,643 (17.9)	284,999 (73.8)
PHEN	3400 (17.7)	1521 (10.4)	33,773 (27.2)	10,841 (3.4)	101,712 (26.3)
Qsymia	347 (1.8)	331 (2.3)	397 (0.3)	334 (0.1)	9434 (2.4)

Data presented as mean ± SD or n (%).

For the unexposed comparator group, 73.8% had previously used TPM, 26.3% had previously used PHEN, and only 2.4% had previously used fixed-PHEN/TPM. Current users of any of the medications were less likely than the unexposed to have epilepsy. The prevalence of comorbidities and other medication use among the unexposed cohort was most similar to that among current TPM users. Both the unexposed cohort and current users of TPM had a greater baseline history of stroke, transient ischemic attack, migraine, and epilepsy compared with the current users of PHEN/TPM or PHEN.

Most patients initiating PHEN/TPM (76%) were fixed-PHEN/TPM users. Compared with the unexposed cohort, patients initiating PHEN/TPM were older and more likely to have a recorded history of obesity. In addition, patients initiating PHEN/TPM were more likely than the unexposed cohort to have hypertension, hyperlipidemia, diabetes, and sleep apnea.

### Unadjusted incidence rates

The number of events, person-time of follow-up, unadjusted incidence rates, and 95% confidence intervals (CIs) for MACE and its components (hospitalization for AMI or stroke and in-hospital CV-related death) are listed in Table 2. The unadjusted incidence rate of MACE among current users of PHEN/TPM and fixed-PHEN/TPM was lower than the rate of MACE among the unexposed cohort. However, the number of events was small, producing considerable statistical variability (as evidenced by wide 95% CIs). The current use of PHEN was associated with lower rates of MACE compared with the unexposed cohort. The current use of TPM was associated with greater rates of MACE compared with that in the unexposed cohort.

**Table 2.** Crude Incidence Rates Per 1000 Person-Years and 95% CIs for MACE and Components of This Outcome

Variable	Current Use Periods				Unexposed (n = 386,136)
	PHEN/TPM (n = 19,184)	Fixed-PHEN/TPM (n = 14,586)	PHEN (n = 124,334)	TPM (n = 316,388)	
Person-years	3245	2587	24,107	64,607	310,665
MACE					
Events, n	3	1	22	218	622
Events/1000 person-years	0.92 (0.19–2.70)	0.39 (0.01–2.15)	0.91 (0.57–1.38)	3.37 (2.94–3.85)	2.00 (1.85–2.17)
AMI					
Events, n	1	0	11	62	335
Events/1000 person-years	0.31 (0.01–1.72)	0.00 (0.00–1.43)	0.46 (0.23–0.82)	0.96 (0.74–1.23)	1.08 (0.97–1.20)
Stroke					
Events, n	2	1	10	154	258
Events/1000 person-years	0.62 (0.07–2.23)	0.39 (0.01–2.15)	0.41 (0.20–0.76)	2.38 (2.02–2.79)	0.83 (0.73–0.94)
CV-related death					
Events, n	0	0	1	2	29
Events/1000 person-years	0.00 (0.00–1.14)	0.00 (0.00–1.43)	0.04 (0.00–0.23)	0.03 (0.00–0.11)	0.09 (0.06–0.13)

Data in parentheses are 95% CIs.

The incidence rates of MACE were greater among both current users of TPM and the unexposed cohort relative to other current-use cohorts. The rates of AMI and stroke were greater than the rates for in-hospital CV death in all current-use and unexposed periods. The average length of the current-use periods for all medications ranged from 2.1 to 2.5 months.

### Results from adjusted analyses of MACE and individual components

Propensity score adjustment created a reasonable balance between the current-use periods and unexposed periods for all variables included in the propensity score models. The number of events and person-years of follow-up after propensity score stratification and trimming and the adjusted IRRs, IRDs, and 95% CIs for all outcomes are listed in Table 3. After propensity score adjustment, the rates of MACE among current users of PHEN/TPM, fixed-PHEN/TPM, and PHEN remained lower than those among the unexposed cohort, and the rate of MACE among current users of TPM remained greater than that among the unexposed cohort. Compared with the crude IRRs and IRDs, the propensity score-adjusted measures were closer to the null (IRR: PHEN/TPM, 0.57; 95% CI, 0.19 to 1.78; fixed-PHEN/TPM, 0.24; 95% CI, 0.03-1.70; PHEN, 0.56; 95% CI, 0.34 to 0.91; TPM, 1.58; 95% CI, 1.33 to 1.87). No substantial differences were found in the IRR and IRD between the unadjusted and adjusted results, indicating that the net amount of confounding was modest.

**Table 3.** Adjusted IRRs and IRDs for MACE and Components of This Outcome

Variable	PHEN/TPM		Fixed-PHEN/TPM		PHEN		TPM	
	Current Use	Unexposed (Reference)	Current Use	Unexposed (Reference)	Current Use	Unexposed (Reference)	Current Use	Unexposed (Reference)
Person-years	2820	232,470	2207	217,665	22,218	251,807	60,889	291,147
MACE								
Events, n	3	424	1	395	17	423	186	539
IRR (95% CI)	0.57 (0.19 to 1.78)		0.24 (0.03 to 1.70)		0.56 (0.34 to 0.91)		1.58 (1.33 to 1.87)	
IRD (95% CI)	−0.79 (−2.03 to 0.44)		−1.43 (−2.37 to −0.50)		−0.62(−1.02 to −0.22)		1.11 (0.64 to 1.57)	
AMI								
Events, n	1	240	0	225	9	241	51	296
IRR (95% CI)	0.35 (0.05 to 2.52)		0.00 (0.00 to NC)		0.51 (0.26 to 1.00)		0.79 (0.59 to 1.07)	
IRD (95% CI)	−0.66 (−1.37 to 0.06)		−1.02 (−1.20 to −0.85)		−0.39 (−0.68 to −0.10)		−0.22 (−0.48 to 0.04)	
Stroke								
Events, n	2	167	1	154	7	167	133	217
IRR (95% CI)	0.89 (0.22 to 3.53)		0.55 (0.08 to 3.85)		0.58 (0.27 to 1.24)		2.81 (2.26 to 3.50)	
IRD (95% CI)	−0.09 (−1.10 to 0.92)		−0.37 (−1.29 to 0.54)		−0.23 (−0.49 to 0.03)		1.38 (1.01 to 1.76)	
CV-related death								
Events, n	0	17	0	16	1	15	2	26
IRR (95% CI)	0.00 (0.00 to NC)		0.00 (0.00 to NC)		1.03 (0.12 to 8.67)		0.35 (0.08 to 1.45)	
IRD (95% CI)	−0.04 (−0.07 to −0.02)		−0.04 (−0.06 to −0.02)		0.00 (−0.09 to 0.09)		−0.06 (−0.12 to 0.00)	

Data are adjusted for propensity score decile after trimming.

Abbreviation: NC, not calculated.

Just as with the crude results, the rates of AMI and stroke during the current-use periods of PHEN/TPM and fixed-PHEN/TPM were lower than those during the unexposed periods. The current users of PHEN had lower rates of AMI (IRR, 0.51; 95% CI, 0.0.26 to 1.00) and stroke (IRR, 0.58; 95% CI, 0.27 to 1.24) compared with the unexposed cohort. In contrast, the rate of CV death was similar for current users and the unexposed cohort (IRR, 1.03; 95% CI, 0.12 to 8.67). The current users of TPM had greater rates of stroke (IRR, 2.81; 95% CI, 2.26 to 3.50) and lower rates of AMI (IRR, 0.79; 95% CI, 0.59 to 1.07) and CV death (IRR, 0.35; 95% CI, 0.08 to 1.45) compared with the unexposed cohort. Event numbers, especially for CV death, were small, and the 95% CIs around the effect measures for components of MACE were wide for all medication cohorts.

### Sensitivity and bias analyses

We conducted several sensitivity and bias analyses among the PHEN cohort and TPM cohort, both of which had >10 MACE outcomes during the current-use periods. These sensitivity analyses varied with the time after last drug dispensing to the end of the exposure period, extended the required time period without the drug before initiating subsequent use, limited the current-use period to only the first 6 months of medication use, and assessed the effect of assessing only in-hospital death. The results were qualitatively similar to the primary results for each of these analyses.

Sensitivity analyses also were conducted with mutually exclusive, current-use medication cohorts, and unexposed periods among former users of PHEN, TPM, and PHEN/TPM. The results of these analyses were directionally similar to the results listed in Table 3, but in all cases were closer to the null (Table 4). For example, the IRR for the primary results for current users of PHEN/TPM vs unexposed former users of any medication was 0.57 (95% CI, 0.19 to 1.78), and the IRR for the mutually exclusive current-use vs unexposed PHEN/TPM comparison was 0.93 (95% CI, 0.20 to 4.32). The number of events was smaller for each of these analyses than those in the primary results, leading to wider 95% CIs.

**Table 4.** Sensitivity Analysis: Event Counts, Person-Time, Propensity Score–Adjusted Incidence Rate Ratio, and Incidence Rate Difference for MACE Comparing Current Use and Unexposed Former Users of Each (Mutually Exclusive) Medication

Cohort	Events, n	Person-Years	IRR (95% CI)	IRD (95% CI)
PHEN/TPM				
Current use	2	2901.9	0.93 (0.20 to 4.32)	−0.06 (−1.19 to 1.08)
Unexposed	7	9146.1	Reference	Reference
PHEN				
Current use	17	18,636.2	0.78 (0.46 to 1.30)	−0.27(−0.77 to 0.23)
Unexposed	93	77,797.2	Reference	Reference
TPM				
Current use	188	55,714.3	1.49 (1.26 to 1.77)	1.11 (0.58 to 1.63)
Unexposed	457	201,561.8	Reference	Reference

Each current-use group was compared with former users of the same medication.

## Discussion

The rationale for including the unexposed periods among former users rather than nonusers as the referent group was that the CV risk is expected to be greater in an obese population than in a nonobese population. However, it was not practical to identify an untreated obese population in an administrative claims database because of inconsistent coding of this condition. By restricting the comparison rates to periods after the use of PHEN, TPM, or PHEN/TPM, we identified a population that was not currently exposed to the medications of interest (PHEN, TPM) but was expected to have similar CV risk to the obese population currently using these medications. Although the study did not use self-matching of different periods, many subjects contributed to both the treated and the untreated cohorts, achieving partial self-matching with its control of factors that remain constant over time in an individual.

We controlled for confounding from the differences that remained between the treatment and comparator groups using propensity score modeling and stratification. Sensitivity analyses provided further insight into the robustness of the results to the decisions made during the design of the study. The results were similar for PHEN and TPM when the MACE outcome was modified to include heart failure, when assuming that only a portion of deaths were captured during current-use periods, when assessing only the first 6 months of each current-use or unexposed period, and when assessing other potential confounders (*e.g.,* proxy for smoking). The results from sensitivity analyses were generally closer to the null, and, because of the more restricted follow-up time, the 95% CIs were wider. In these analyses, the crude results were not appreciably different from the adjusted results, suggesting little confounding. Comparisons of mutually exclusive current-use periods vs unexposed periods among each medication cohort led to few events for PHEN/TPM (<10 MACE events total) and wide 95% CIs.

The present study included a large number of patients treated for several years within a US claims data source representative of the patient experience with PHEN/TPM in the United States. The FDA has acknowledged that observational studies using databases are an effective method to generate information on the safety of medications as used in usual clinical practice within a much shorter time than would be possible with a prospective study (11). The present study was also prespecified via protocol and conducted in accordance with both regulatory and international society guidelines for observational database studies (12–15). It included outcome measures previously validated within claims data. In an era in which regulators are calling for increased use of real-world evidence for regulatory decision-making, the present database analysis has provided timely data on a large number of patients in a manner that is actionable (*e.g.,* can rule out doubling of MACE outcomes among users of PHEN/TPM). Furthermore, the insights gained from the present study were obtained within several months, rather than over several years.

The reason for the greater risk of stroke among current TPM users compared with former TPM users is not obvious. It might be a real difference, a chance finding, or an unidentified bias. The incidence rates of MACE, driven by the rates of stroke, were greater for both current users of TPM and the unexposed after the use of TPM compared with other medications. The exclusion criteria specific to TPM imply that these patients do not constitute all patients prescribed TPM. Furthermore, the exclusion criteria specific to TPM were designed to remove patients with epilepsy from the study, because they have an increased risk of stroke. However, more patients with a history of antiepileptic drug use were present in the TPM cohort than in the PHEN cohort (27% vs 12%) (16).

Perhaps the subset of TPM users included were at an increased risk of this outcome in a way that was inadequately measured in the present study (*e.g.,* they might have had increased systolic blood pressure and were thus prescribed TPM because of its known effect of decreasing systolic blood pressure and/or the attempt to control for an epilepsy diagnosis and medication use was insufficient) (17, 18).

Data for some of the potential confounders of interest (*e.g.,* smoking, heart rate, race/ethnicity) were not available from the administrative claims database. The bias analysis demonstrated, however, that any unmeasured confounder would have had to be strongly unbalanced between the cohorts to have had a meaningful confounding effect.

Although the fixed-dose combination of PHEN/TPM has been approved for chronic, long-term use, the average duration of use for current users was only 2.1 months. This figure was comparable with the average duration of use of PHEN (2.3 months) and TPM (2.5 months). These durations of use were shorter than those in the premarket clinical trials but presumably reflect actual clinical patterns of use and might further decrease any concerns about the risk of CV outcomes.

## Conclusion

The present analysis of PHEN used concurrently with TPM, either separately or in fixed-dose combination, provides some reassurance about the absence of large risks of CV outcomes caused by these agents as used in clinical practice. We found a trend for a lower rate of MACE and other CV outcomes among those with current exposure to PHEN/TPM (including the fixed-dose combination) than among the unexposed cohort. However, considerable statistical uncertainty remains, stemming from the small number of events, yielding 95% CIs that ranged from strong negative associations to small positive associations, with an upper 95% confidence limit below a doubling of the rate for the composite MACE outcome, during the relatively short time patients were taking the medication.

## Abbreviations:

AMI: acute myocardial infarction
CV: cardiovascular
FDA: Food and Drug Administration
ICD-9-CM: *International Classification of Diseases, Ninth Revision, Clinical Modification*
IRD: incidence rate difference
IRR: incidence rate ratio
MACE: major adverse cardiovascular events
PHEN: phentermine
TPM: topiramate
